# The Association of Plasma Selenium and Selenoprotein P Levels with Depression Severity and Anxiety Symptoms Among Medical Students in Latvia

**DOI:** 10.3390/medicina61010003

**Published:** 2024-12-24

**Authors:** Zanda Birģele, Paula Marija Vimba, Anastasija Ševčenko, Andrejs Šķesters, Gunta Ancāne, Laura Valaine

**Affiliations:** 1 Clinic of Psychosomatic medicine and Psychotherapy, Riga Stradiņš University, LV-1046 Riga, Latvia; 2 Faculty of Medicine, Riga Stradiņš University, LV-1007 Riga, Latvia; 3 Institute of Occupational Safety and Environmental Health, Riga Stradiņš University, LV-1067 Riga, Latvia; andrejs.skesters@rsu.lv; 4 Department of Psychosomatic Medicine and Psychotherapy, Riga Stradiņš University, LV-1046 Riga, Latvia; gunta.ancane@rsu.lv (G.A.); laura.valaine@rsu.lv (L.V.)

**Keywords:** oxidative stress, selenium, selenoprotein P, depression, anxiety

## Abstract

*Background and Objectives*: Oxidative stress has been identified as a key process involved in different diseases, particularly depression. Selenium (Se) protects against oxidative stress, one of the pathogenic mechanisms involved in affective disorders. Selenium is incorporated into antioxidant selenoproteins, such as selenoprotein P, which acts as the main selenium-transport protein in plasma and as an extracellular oxidant defense mechanism. This study aimed to determine whether lower selenium and selenoprotein P levels correlate with high levels of depression and anxiety symptoms. *Materials and Methods*: The research design was a quantitative cross-sectional study among employed fourth-year medical students at Riga Stradins University in Latvia. The respondents were selected using convenience samples. The symptoms of anxiety were assessed using the Generalized Anxiety Disorder-7 (GAD-7) scale, and the symptoms of depression were assessed using the Patient Health Questionnaire-9 (PHQ-9) scale. *Results*: A total of 32 respondents participated; 90.6% (*n* = 29) were female. A significant association was found between selenoprotein P and symptoms of depression (*p* = 0.006), as well as between selenoprotein P and symptoms of anxiety (*p* = 0.012). The median selenium level was not significantly lower (*p* = 0.214) in the study group compared to the control group. *Conclusions*: There is a statistically significant correlation between selenoprotein P and symptoms of depression and anxiety, and there is a tendency for students with symptoms of depression and anxiety to have lower selenium levels. However, alternative unrecognized oxidative stress mechanisms involved in the development of symptoms of depression and anxiety, involving selenium and selenoprotein P pathways, may exist. Consequently, further research assessing possible alternative pathways and the effect size is required.

## 1. Introduction

The number of people with anxiety and depression symptomatology is growing rapidly worldwide [1,2]. Although there is no definite hypothesis on the pathophysiology of anxiety and depression, it is known that the abnormal functioning of the stress response, inflammatory processes, neurogenesis, neurotransmission, and synaptic plasticity underlie the pathophysiology of these disorders [3]. The role of oxidative stress in depression is well recognized and plays an important role in the onset and development of the disease. More specifically, oxidative modifications to proteins have been proposed as a potential factor in the onset and progression of several psychiatric disorders, including anxiety and depressive disorders [4,5]. Oxidative stress is defined as an imbalance between the production of oxidants (free radicals or reactive oxygen species) and their eradication by protective mechanisms, such as antioxidants, including selenium [6].

Selenium is a micronutrient component of several biologically active selenoproteins, such as glutathione peroxidases and thioredoxin reductases. In addition, selenoprotein P is involved in several aspects of brain functioning and may exert antidepressant and anxiolytic effects through multiple pathways [7,8]. Selenium is also important for protection against oxidative stress. Se supplementation might reduce oxidative stress by increasing the total antioxidant capacity and glutathione peroxidase levels and decreasing serum plasma malonaldehyde [9,10].

However, there is no conclusive evidence about the relevance of selenium and selenoprotein P levels in relation to symptoms of depression and anxiety [11,12]. Moreover, selenium has been proven to be crucial to the brain structure, albeit it may also be neurotoxic, subject to the dosage and speciation [13].

Selenoprotein P is a secreted glycoprotein that contains most of the selenium in plasma and serves as the main selenium-transport protein in mammals. Additionally, selenoprotein P has been implicated as an oxidant defense mechanism in the extracellular space and plays an important role in the transport of selenium to the brain for the synthesis of essential selenoproteins. Further, studies have illustrated that selenoprotein P is not limited solely to selenium transport, as it is thought to exhibit certain antioxidant properties, contributing to redox regulation [13,14].

The prevalence of psychological stress in undergraduate medical students across multiple nationalities has been estimated to be 10–96% [15], which is greater than in non-medical students and the general population. In addition, depression and anxiety are one of the most common health problems for university students. A systematic review estimated that the mean prevalence of depressive disorders in university students was 30.6%, which is considerably higher than the rates reported in general populations [16]. Mental health among university students worldwide is a significant public health concern, particularly for medical students. Research involving medical school graduates has demonstrated that psychological distress can lead to a reduced quality of patient care, compromised patient safety, and diminished professionalism throughout their subsequent careers [15,17,18,19,20,21,22,23,24].

Medical students are continuously exposed to psychosocial stressors during their studies, which, in turn, can lead to a variety of biological (i.e., oxidative stress), physiological, and psychological imbalances and/or pathologies. In particular, neurotic and anxious students tend to be exposed to greater levels of oxidative stress, in addition to the rigors of medical student training, which contribute to the high level of psychobiological toxicity factors. For example, Sivonova et al. investigated the connection between possible psychological stress caused by undergraduate examinations at university and oxidative stress, where the results suggested that during university examinations, students experience increased oxidative stress, and that mental stress contributes to oxidative stress in the body [25,26].

These significant psychobiological toxicity factors contribute to the significant stress among students in medical schools. The state of psychological stress is associated with increased oxidative stress, which generates a large number of free radicals that are capable of initiating or promoting oxidative injury and a positive relationship between work-related risk factors, including physiological and psychological stress [26,27].

Consequently, we aimed to further explore the link between oxidative stress mechanisms and mental health, by assessing whether blood plasma levels of selenium and selenoprotein P correlate with symptoms of depression and anxiety in employed, fourth-year working medical students.

## 2. Materials and Methods

### 2.1. Ethics

This study received the endorsement of the Research Ethics Committee at Riga Stradins University, Latvia, under protocol number 2-PĒK-4/48/2023, dated 9 January 2023. To facilitate the collection of blood samples from participants, it was required that they provided telephone numbers in the initial questionnaire. These contact details were essential for subsequent outreach related to the survey. Participants were not required to provide their name, surname, or other personal information that could identify a person.

Participation in the study was entirely voluntary. Participants were provided with informed consent forms alongside detailed explanations regarding the study’s objectives and methodology. Consent for participation in a paper version was acquired from all participants. Furthermore, participants retained the prerogative to exit the study at any point, without the necessity of providing any rationale.

The integrity of participant data confidentiality and protection was upheld in alignment with Regulation (EU) 2016/679 of the European Parliament and the Council (dated 27 April 2016): concerning the protection of natural persons, the processing of personal data, the free movement of such data, effectively superseding Directive 95/46/EC (General Data Protection Regulation), alongside other pertinent statutes and directives. The utilization of personal data, specifically telephone numbers and email addresses, was confined exclusively to communication requisites for the advancing stages of the study (notably, the dissemination of subsequent questionnaires). The anonymity and confidentiality of participants were rigorously maintained following the study’s methodological stipulations. Upon the conclusion of the research, all data were archived in an anonymized format, and all contact information collected in the questionnaire was permanently deleted.

### 2.2. Study Design and Sampling

The study among employed 4th-year working medical students of Riga Stradins University in Latvia was conducted starting from December 2022 to January 2023, and research was conducted in two phases.

On 18 December 2022, students were informed that such a study was planned and would begin after receiving the permission of Ethics Committee. The 4th-year medical student group leaders and Riga Stradins University students’ Facebook group administrators were contacted, and the procedure for informing medical students was clarified. After receiving the approval of the Ethics Committee on 9 January 2023, a Google Forms questionnaire was sent to students on 10 January 2023.

For the participants willing to take part in the study, an online self-reported questionnaire with four parts was used: questions assessing demographic data and diet, and standardized instruments for depression and anxiety, specifically the Patient Health Questionnaire-9 (PHQ-9) and General Anxiety Disorder-7 (GAD-7). Additionally, respondents were asked to provide consent to participate in the study and their telephone numbers for further communication regarding selection for the subsequent phase.

Upon completion of these questionnaires, participants were screened based on the inclusion and exclusion criteria. Of the 58 participants who completed the survey, 40 were selected for further participation based on exclusion criteria. After contacting these 40 individuals, 32 consented to engage in the next phase of the research.

When the data were collected and the second phase of the study was initiated, during 23–27 January 2023, blood tests were performed, where ethical adherence was ensured by obtaining informed consent from all participants through signed physical agreement forms.

### 2.3. Inclusion and Exclusion Criteria

The inclusion criteria required that participants be currently enrolled in the fourth medical study year at Riga Stradins University, actively employed, and have explicitly consented to partake in the study to ensure a consistent academic and professional profile among participants. The exclusion criteria were consumers of any dietary supplements containing selenium, and individuals unwilling to participate.

Participants were divided into two groups based on their PHQ-9 scores: the inclusion criterion for the control group was a PHQ-9 score of <10, and the inclusion criterion for the study group was a PHQ-9 score of ≥10, with 16 participants in each group.

### 2.4. Measurement Tools and Procedures

#### 2.4.1. Initial Phase

In the initial phase, participants reported demographic data, dietary habits, and nutrition-related factors, as well as two standardized questionnaires that assessed the symptoms of anxiety (GAD-7) and depression (PHQ-9).

Demographic data such as age (years), sex (male/female), educational background (study institution: Riga Stradins University (RSU), Latvian University (LU), Riga Technical University (RTU); study year (1st, 2nd, 3rd, 4th, 5th, 6th), and socioeconomic status (working/not working) were reported.

Further dietary habits and nutrition were detected: if they use dietary supplements containing Se (yes/no); how many meals per day are eaten (1/2/3/4 or more); dietary patterns (always eat breakfast/never eat breakfast/sometimes eat breakfast; vegan/vegetarian; consume dairy products/do not consume dairy products).

Symptoms of depression were assessed using the Patient Health Questionnaire-9 (PHQ-9). The PHQ-9 includes nine items, with each corresponding to one of the DSM-IV criteria for depression, and participants rate the frequency of each symptom over the past two weeks on a scale from 0 (not at all) to 3 (nearly every day). A validated Latvian version of the nine-item Patient Health Questionnaire (PHQ-9; range, 0–27) was used to assess the symptoms of depression, Cronbach’s alpha 0.84. The results of the questionnaire were categorized as a dichotomous variable. The cut-off score for clinically significant depression was 10 [27,28].

Symptoms of generalized anxiety were measured using the Generalized Anxiety Disorder-7 (GAD-7) scale. The GAD-7 consists of seven items, with respondents indicating how often they have been bothered by each symptom over the past two weeks, using a similar 0 to 3 scale. A validated Latvian version of the seven-item Generalized Anxiety Disorder (GAD-7) scale (range 0–21) was used to assess symptoms of anxiety, Cronbach’s alpha 0.87 [29,30,31]. The results of the questionnaire were categorized as a dichotomous variable. The cut-off score for clinically significant generalized anxiety was 10 [22,32,33].

#### 2.4.2. Collection and Storage of Plasma Samples

Blood samples were collected using anticoagulant-coated Vacutainer tubes (EDTA), drawing 20 mL of peripheral venous blood from each subject. Blood samples were immediately stored at −20 °C post collection, followed by centrifugation at 3000 rpm for 15 min at 5 °C. Plasma was then aliquoted and stored at −80 °C in Eppendorf tubes to preserve sample integrity for subsequent analyses.

#### 2.4.3. Determination of Blood Plasma Selenium Concentration

The analytical procedure for assessing selenium concentrations in plasma utilizes manual, fluorimetric methodology, with samples prepared using either Li-heparin or EDTA as anticoagulants. This method, identified by the technology code La/Kim 237, employs the “Cary Eclipse” fluorometer (Varian, Inc., Middelburg, The Netherlands) for measurement. The reagents integral to this process include 2,3-diaminonaphthalene, sodium ethylenediaminetetraacetic acid (Na-EDTA), 30% hydrogen peroxide, hydroxylammonium chloride, nitric acid, ammonia, formic acid, hydrochloric acid, sulfuric acid, chloric acid, and n-hexane. The underlying principle of this method involves the reduction of selenium into tetravalent compounds. These compounds subsequently form complexes with 2,3-diaminonaphthalene, the fluorescence of which is measured at an excitation wavelength of 369 nm and an emission wavelength of 518 nm, with a peak detection range of 518–515 nm [34]. The reference interval established for selenium concentration in plasma is set between 80 and 120 µg/L, providing a standard benchmark for evaluating physiological and pathological states.

Plasma selenium concentrations were quantified using a rigorous multi-day analytical protocol. Initially, 0.5 mL of plasma was treated with 1 mL of concentrated HNO_3_ and glass beads, allowing the mixture to macerate at room temperature for 24 h. This was followed by 0.4 mL conc. And the addition of HClO_4_/H_2_SO_4_ (*v*:*v* 20:1) and controlled heating cycles: 120 °C for 20 min, 150 °C for 60 min, and 90 min at 180 °C. After cooling, 0.1 mL of H_2_O_2_ 30% was added to the samples and heated at 150 °C for 10 min; in the last step, 1 mL of 6 M HCl was added and heated at 120 °C for 10 min. After cooling the samples, the pH was set within the range of 1.5–2.0. Then, 1 mL of 6 M formic acid, 1.5 mL of Na_2_EDTA/Hydroxylamine-HCl (*v*/*v* 1:5), and 1 mL of 2,3-Diaminophthalene (6.3 mM in 0.1 M HCl) were added to the samples, which were then heated at 50 °C for 30 min and cooled rapidly. Next, 2.5 mL of n-Hexane was added, and the mixture was shaken for 2 min at 3000 vibrations per minute, followed by centrifugation at 3000 rpm for 10 min. The resulting fluorophore was extracted into hexane, and its fluorescence was measured (Ex 369 nm/Em 518 nm).

As internal standards, we used (a) Sero Norm Trace Element Serum L-1 (Sero AS, Billingstad, Norway) [35] and (b) Sigma-Aldrich Selenium Standard for AAS, Cat. From 89498-250 mL, Steinheim, Germany [36].

#### 2.4.4. Determination of Blood Plasma Selenoprotein P Concentration

Selenoprotein P levels were quantified using a commercially available ELISA kit: Human Selenoprotein P (SEPP1) ELISA kit, Cat. No. CSB-ELO021018HU, CUSABIO, the USA, following the manufacturer’s protocol [37]. This involved specific reagent preparations, sample incubations, and optical density measurements at a wavelength specified by the kit instructions. Results were read by the microplate analyzer SPAR, Tecan, Austria.

### 2.5. Statistical Analysis

Data were statistically analyzed using IBM SPSS Statistics 29.0.0.0. Non-parametric tests, including Spearman correlation, Mann–Whitney U-test, and chi-square test, were employed to discern significant associations and differences. A *p*-value of less than 0.05 was adopted as the threshold for statistical significance.

## 3. Results

### 3.1. Descriptive Statistics of the Study Population

Plasma samples were obtained from 32 subjects. The demographic composition of the sample was predominantly female, accounting for 90.6% (*n* = 29) of the participants, with males constituting the remaining 9.4%. All the participants were employed students currently studying in the fourth year of Medicine at Riga Stradins University (Table 1).

The depression and anxiety scores differed between the study and control groups, as shown by the Patient Health Questionnaire-9 (PHQ-9) and Generalized Anxiety Disorder-7 (GAD-7) results. The study group’s median PHQ-9 score was 15.0 (IQR 10.3–19.8), suggesting moderate to moderately severe depression, and their median GAD-7 score was 13.0 (IQR 9.3–14.8), indicating moderate anxiety. These figures were substantially higher than those of the control group, with median scores of 4.5 (IQR 3.0–7.8) for PHQ-9 and 4.5 (IQR 3.3–7.0) for GAD-7, pointing to minimal depression and anxiety (see Table 2). Additionally, our analysis revealed a significant and strong correlation between depression and anxiety levels (*p* < 0.001, R = 0.84), highlighting a potential close relationship between these two mental health conditions.

We compared selenium levels with selenoprotein P levels using Spearman’s correlation and established a solid positive monotonic relationship between the two variables (ρ = 0.974), which suggests that higher selenium levels are consistently associated with higher selenoprotein P levels.

### 3.2. Correlation Between Selenium and PHQ-9 and GAD-7 Scores

**Figure 1:**
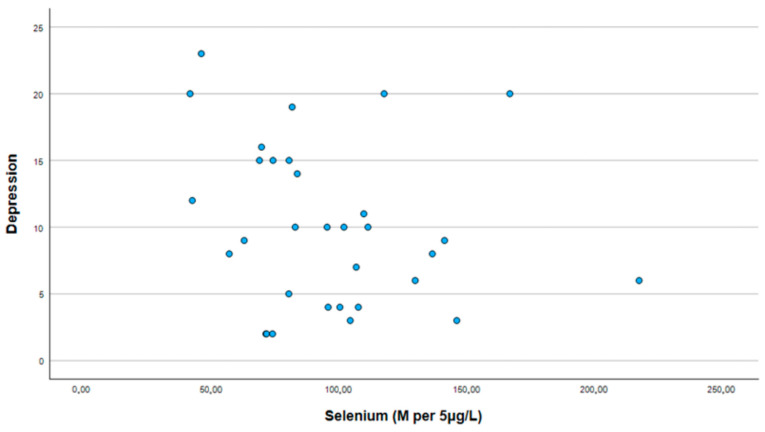
Correlation between selenium and PHQ-9 score (symptoms of depression).

**Figure 2:**
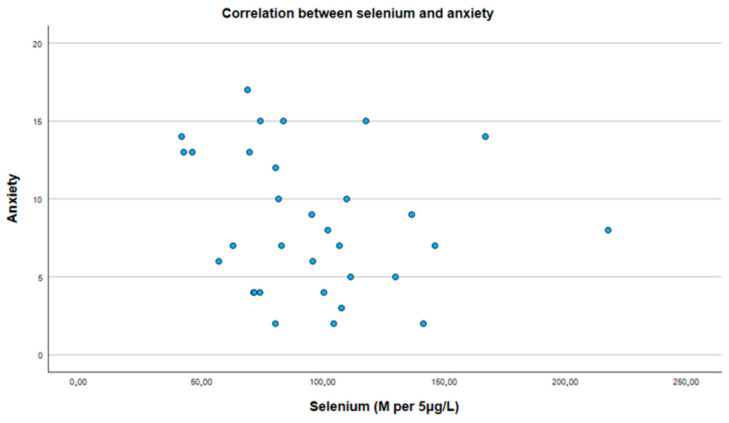
Correlation between selenium and GAD-7 score (symptoms of anxiety).

The median selenium level was slightly lower in the study group, at 81.7 per 5 μg/L, compared to 101.8 per 5 μg/L in the control group. However, our analysis did not reveal a significant correlation between selenium levels and scores in the PHQ-9 scale (see Figure 1), which was used for symptoms of depression (r = −0.202, *p* = 0.267), or scores in GAD-7 (see Figure 2), which was used for symptoms of generalized anxiety disorder (r = −0.199, *p* = 0.275), within the study population (see Table 3).

**Figure 3:**
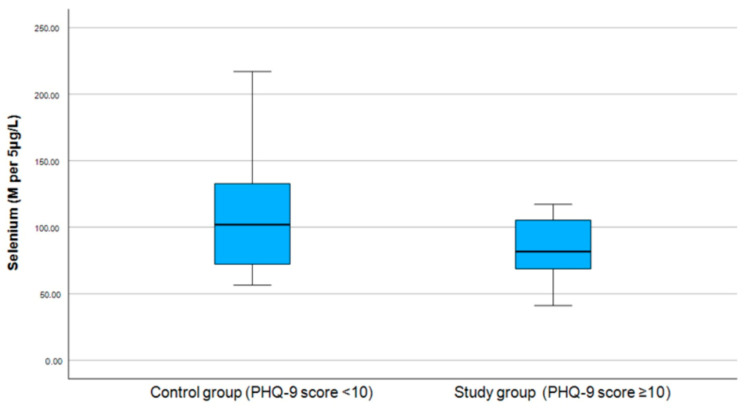
Correlation between selenium and PHQ-9 score.

**Figure 4:**
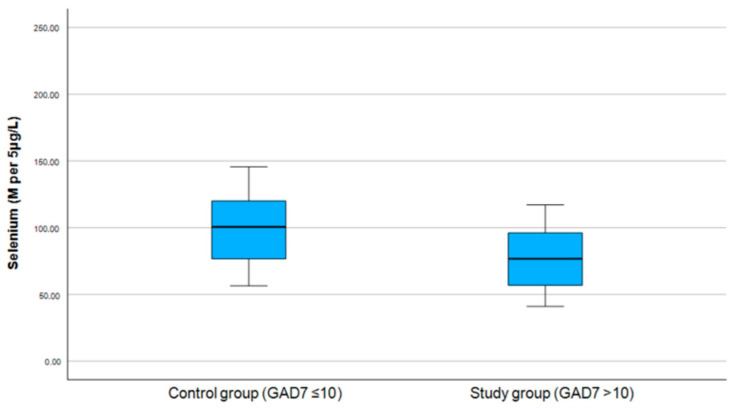
Correlation between selenium and GAD-7 score.

For further analysis, the selenium levels were classified as follows: within the normal range (80–120 per 5 μg/L), above the normal range (>120 per 5 μg/L), and below the normal range (<80 M per 5 μg/L). The results shown in Table 4 indicate no significant differences in selenium range group levels between the study and control groups (see Figure 3 and Figure 4).

### 3.3. Correlation Between Selenoprotein P and PHQ-9 and GAD-7 Scores

**Figure 5:**
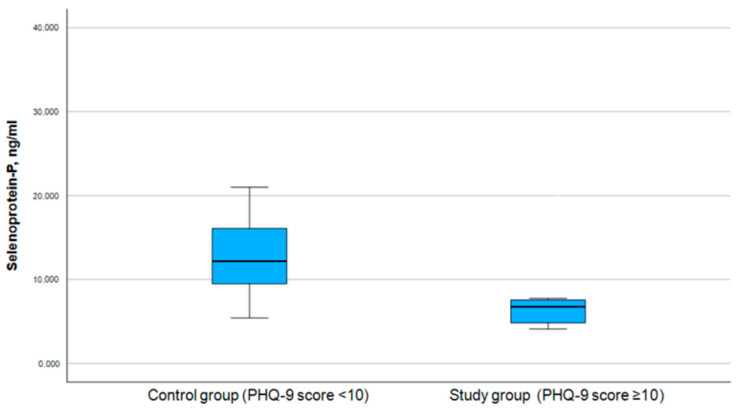
Correlation between selenoprotein P and PHQ-9 score.

**Figure 6:**
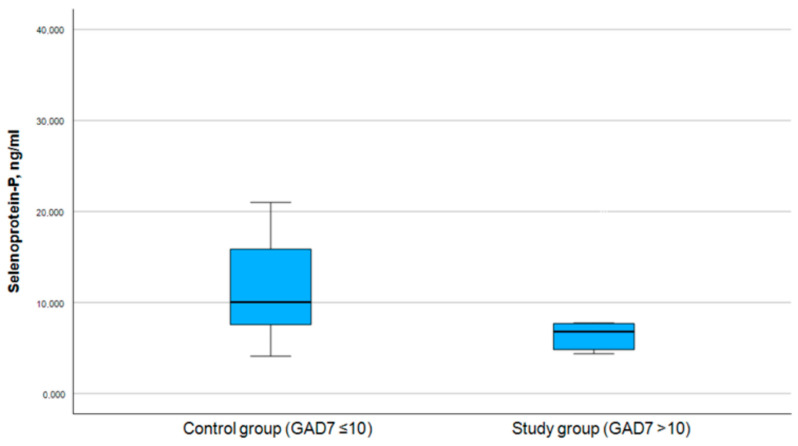
Correlation between selenoprotein P and GAD-7 score.

Similarly to selenium, selenoprotein P data were categorized into distinct groups for further analysis, where the selenoprotein P levels were divided into those above and below the mean value (8.1 ng/mL) (see Table 5). The Pearson chi-square test was used for the association between levels of selenoprotein P and the PHQ-9 score (symptoms of depression) and GAD-7 score (symptoms of anxiety) (see Table 6), which were divided into study and control groups accordingly (see Figure 5 and Figure 6). 

**Figure 7:**
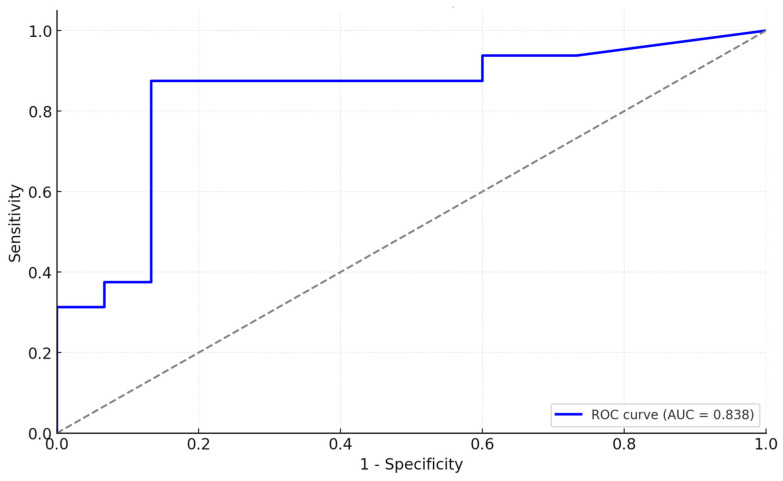
ROC curve for selenoprotein levels in individuals with symptoms of depression.

**Figure 8:**
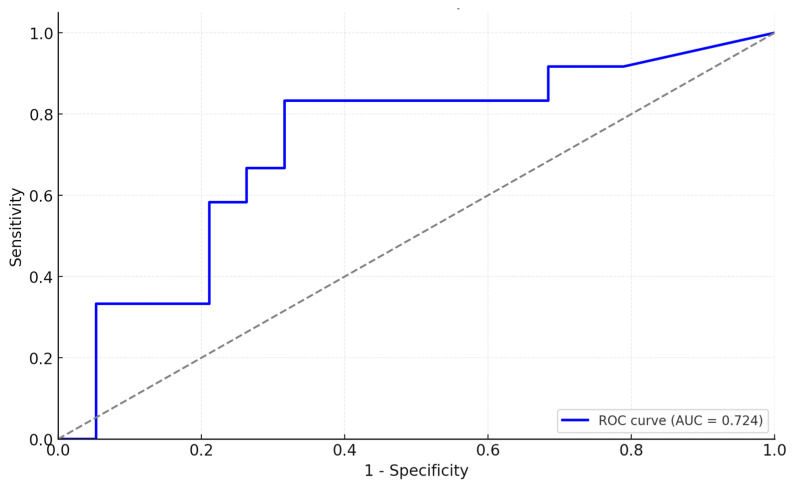
ROC curve for selenoprotein levels in individuals with symptoms of anxiety.

For a better understanding of the collected data, we used the ROC curve analysis for symptoms of depression, revealing an area under the curve (AUC) of 0.838 (SE = 0.078, *p* < 0.001, 95% CI [0.685, 0.990]) (see Figure 7). This substantial AUC underscores the high diagnostic accuracy of selenoprotein levels in identifying individuals with symptoms of depression. In contrast, for generalized anxiety symptoms, the AUC was moderately high at 0.724 (SE = 0.097, *p* = 0.039, 95% CI [0.533, 0.914]) (see Figure 8), indicating a moderate accuracy in diagnosing symptoms of generalized anxiety disorders based on selenoprotein levels.

The Pearson chi-square test for the association between selenoprotein levels and depression symptom categories indicated a significant association between selenoprotein levels and the presence of depression symptoms (chi-square = 17.052, df = 1, *p* < 0.001). The results suggest that lower selenoprotein levels are significantly associated with clinically significant symptoms of depression. Similarly, a significant association was observed between selenoprotein levels and generalized anxiety symptom categories (chi-square = 7.888, df = 1, *p* = 0.005), albeit with a lower level of significance compared to depression symptoms. Although the results were statistically significant, the sample size was very limited; thus, caution is needed in interpreting the results.

## 4. Discussion

The results of this study show that there is a tendency for lower selenium levels to be associated with higher depression and anxiety symptom levels, although the correlation is not statistically significant. Given the observed tendency for students with symptoms of depression and anxiety to have lower selenium levels, we suggest that there may be a potential link between selenium pathways and the pathogenesis of these mental health conditions. Further to this potential link, a Brazilian study illustrated a reduction in the occurrence of depression in association with the consumption of high doses of selenium [38]. In addition, it appears that lower serum selenium concentrations were associated with higher anxiety [39]. However, the association between selenium intake and the depression score was not significant [40]. Additionally, an optimal range of serum selenium between ∼82 and 85 μg/L was associated with a reduced risk of depressive symptoms in young adults [41].

Notwithstanding current research, there remains a gap in understanding the specific mechanism by which selenium and selenoproteins protect cells and tissues from cellular damage due to oxidative stress [42]. In this study, selenium and selenoprotein P were found to be strongly linked and correlated (ρ = 0.974). According to Dogaru et. al (2023), selenoproteins are not limited in their role as antioxidative enzymes and are a key element of selenium transportation [43]. As a result, selenoproteins may play an important role in new therapies for different diseases caused by the dietary and general status of selenium [43].

As previous studies have shown, the transport of selenium relies on many different selenoproteins [44], which may have influenced the correlation found in our study. Therefore, the significant correlation observed in this study between selenium and selenoprotein P could be explained as a function of selenium transport facilitated by different selenoproteins, including, but not limited to, selenoprotein P.

As selenium and selenoprotein P are repeatedly shown to interact and correlate with each other [45,46] and further correlate with symptoms of depression and anxiety, it is important to continue exploring these pathways further. Essentially, if selenium levels in an organism can be altered through selenium supplements, supplementation may improve symptoms of depression and anxiety [12].

Our findings align with the hypothesis that alterations in selenoprotein levels could potentially be involved in the pathophysiology of depression and anxiety through previously researched mechanisms related to oxidative stress and inflammation [45].

Our analysis also identified a strong correlation between depression and anxiety levels (*p* < 0.001), underscoring the interconnectedness of these mental health conditions. Various studies show the same strong correlation between anxiety and depression in medical students [46,47], including during pre- and post-COVID-19 times [48].

As this connection is widely recognized in previous studies [46,47,48], it also proves to be true in our study, further acknowledging the strong association between the symptoms of mental illnesses and the connection between symptoms of depression and anxiety.

### Strengths and Limitations

Our findings suggest that while there may be an association between lower selenium levels and poorer mental health outcomes, these results did not provide conclusive evidence to support this hypothesis. The possible correlations between selenium and selenoprotein P with the symptoms of depression and anxiety illustrate the potential of further studies with bigger sample sizes to study the potential relationship between selenium levels and mental health outcomes to a greater extent. It is important to note that selenium is one of many factors that could influence mental health. Therefore, further research is warranted to better understand its specific role in this context.

This study measured selenium and selenoprotein P concentration in blood serum instead of measuring dietary selenium intake among study participants; therefore, the bioavailability of selenium and selenoprotein P, which can differ due to various reasons, was not accounted for.

Although this study considered potential participants consuming selenium supplements, thus excluding them from this study, the overall dietary regime was not further analyzed.

However, considering the fact that the lack of micronutrients is adjustable and alterable, even a slight association with symptoms of anxiety and depression and selenium would be beneficial to the medical community and public health. The findings of this research may have implications for people who consume a diet that is either low or very high in selenium, as well as for consumers of selenium supplements.

Consequently, our findings highlight the need for further research to explore the underlying factors contributing to the observed disparities in mental health, as well as the potential impact of dietary habits on mental health outcomes. Future investigations could explore larger sample sizes, diverse populations, and longitudinal studies to evaluate the relationship between selenium levels and mental health more comprehensively. The consideration of other potential confounding variables, such as dietary habits, lifestyle factors, and genetic predispositions, may provide a more nuanced understanding of this association’s mechanisms.

It is crucial to note that as a pilot study, the sample size in this research was very limited, and this study looked at symptoms of depression and anxiety instead of already established diagnoses of depression and anxiety. Thus, the results are not representative of the overall population, and further repetition with greater samples is mandated before recognizing selenoprotein P as a valid predictor of the pathogenic mechanism underlying symptoms of depression and anxiety.

## 5. Conclusions

As a pilot study, it was observed that a suggestive but inconclusive trend towards lower selenium levels was associated with higher depression symptom scores in the study group. Despite the apparent trend of lower selenium levels among students experiencing depression and anxiety, these associations did not meet the threshold for statistical significance. However, lower selenoprotein P levels correlate with higher depression and anxiety symptom levels, with high diagnostic accuracy of selenoprotein P levels for depression and moderately high accuracy for anxiety, suggesting the potential utility of selenoprotein P as a biomarker for depression and anxiety.

Further research opportunities may exist in yet-unrecognized oxidative stress mechanisms involving selenium and selenoprotein P pathways in the development of different disorders, such as depression and anxiety. However, due to a low participant count, further research is definitely needed to better characterize and understand the complexity of the structure and function of selenium and selenoproteins for the improvement of mental health.

## Figures and Tables

**Table 1:** Baseline characteristics of the study population.

Baseline Characteristic	*n* (%)
Male	3 (9.4)
Female	29 (90.6)
Previously diagnosed anxiety or depression	13 (40.6)
Age (mean (SD)) Selenium (M per 5 μg/L)	22.2 (0.7) 81.7

**Table 2:** Patient Health Questionnaire (PHQ-9) and Generalized Anxiety Disorder-7 (GAD-7) scores.

	Patient Health Questionnaire-9 (PHQ-9)	Generalized Anxiety Disorder (GAD-7)
Study group		
Median score	15.0	13.0
IQR	10.3–19.8	9.3–14.8
Control group		
Median score	4.5	4.5
IQR	3.0–7.8	3.3–7.0

**Table 3:** Correlation between selenium and PHQ-9 and GAD-7 scores.

	GAD-7 Score (Symptoms of Anxiety)	PHQ-9 Score (Symptoms of Depression)
Selenium	*p* = 0.275, r = −0.199	*p* = 0.267, r = −0.202

**Table 4:** Distribution of selenium groups between study and control groups.

Selenium Level	Below the Normal Range, n (% of This Group)	Within the Normal Range, n (% of This Group)	Above the Normal Range, n (% of This Group)	*p*-Value
Control group	7 (53.85)	8 (61.54)	1 (16.67)	0.174
Study group	6 (46.15)	5 (38.46)	5 (83.33)	

**Table 5:** Median selenoprotein P level and correlation between selenoprotein P and symptoms of anxiety.

	Selenoprotein P, ng/mL (Mean)	Anxiety	Depression
Selenoprotein P	8.1 ng/mL	*p* = 0.12	*p* = 0.006

**Table 6:** Pearson chi-square test for the association between levels of selenoprotein P, divided into groups, and the PHQ-9 score (symptoms of depression) and GAD-7 score (symptoms of anxiety), divided into study and control groups accordingly.

	PHQ-9 Score (Symptoms of Depression)			GAD-7 Score (Symptoms of Anxiety)		
	Selenoprotein P Level (Median)	Chi-Square	*p*-Value	Selenoprotein P Level (Median)	Chi-Square	*p*-Value
Control group	10.049			9.534		
		17.052	<0.001		7.888	0.005
Study group	6.770			6.770		

## Data Availability

The data are not available due to privacy.

## References

[1] Kupcova I., Danisovic L., Klein M., Harsanyi S. (2023). Effects of the COVID-19 pandemic on mental health, anxiety, and depression. BMC Psychol..

[2] COVID-19 Mental Disorders Collaborators (2021). Global prevalence and burden of depressive and anxiety disorders in 204 countries and territories in 2020 due to the COVID-19 pandemic. Lancet.

[3] Correia A.S., Cardoso A., Vale N. (2023). Oxidative Stress in Depression: The Link with the Stress Response, Neuroinflammation, Serotonin, Neurogenesis and Synaptic Plasticity. Antioxidants.

[4] Fedoce A.D.G., Ferreira F., Bota R.G., Bonet-Costa V., Sun P.Y., Davies K.J.A. (2018). The role of oxidative stress in anxiety disorder: Cause or consequence?. Free Radic. Res..

[5] Bouayed J., Rammal H., Soulimani R. (2009). Oxidative stress and anxiety: Relationship and cellular pathways. Oxidative Med. Cell. Longev..

[6] Zakeri N., Rezaei kelishadi M., Asbaghi O., Naeini F., Afsharfar M., Mirzadeh E., Naserizadeh S.K. (2021). Selenium supplementation and oxidative stress: A review. PharmaNutrition.

[7] Steinbrenner H., Sies H. (2013). Selenium homeostasis and antioxidant selenoproteins in brain: Implications for disorders in the central nervous system. Arch. Biochem. Biophys..

[8] Rajkumar R.P. (2022). Selenium and Its Compounds in the Treatment of Anxiety and Related Disorders: A Scoping Review of Translational and Clinical Research. Futur. Pharmacol..

[9] Siwek M., Sowa-Kućma M., Dudek D., Styczeń K., Szewczyk B., Kotarska K., Misztakk P., Pilc A., Wolak M., Nowak G. (2013). Oxidative stress markers in affective disorders. Pharmacol. Rep..

[10] Hasani M., Djalalinia S., Khazdooz M., Asayesh H., Zarei M., Gorabi A.M., Ansari H., Qorbani M., Heshmat R. (2019). Effect of selenium supplementation on antioxidant markers: A systematic review and meta-analysis of randomized controlled trials. Hormones.

[11] Wang J., Um P., Dickerman B.A., Liu J. (2018). Zinc, Magnesium, Selenium and Depression: A Review of the Evidence, Potential Mechanisms and Implications. Nutrients.

[12] Sajjadi S.S., Foshati S., Haddadian-Khouzani S., Rouhani M.H. (2022). The role of selenium in depression: A systematic review and meta-analysis of human observational and interventional studies. Sci. Rep..

[13] Solovyev N., Drobyshev E., Bjørklund G., Dubrovskii Y., Lysiuk R., Rayman M.P. (2018). Selenium, selenoprotein P, and Alzheimer's disease: Is there a link?. Free. Radic. Biol. Med..

[14] Burk R.F., Hill K.E. (2009). Selenoprotein P-expression, functions, and roles in mammals. Biochim. Biophys. Acta.

[15] Fawzy M., Hamed S.A. (2017). Prevalence of psychological stress, depression and anxiety among medical students in Egypt. Psychiatry Res..

[16] Ibrahim A.K., Kelly S.J., Adams C.E., Glazebrook C. (2013). A systematic review of studies of depression prevalence in university students. J. Psychiatr. Res..

[17] Alvi T., Assad F., Ramzan M., Khan F.A. (2010). Depression, anxiety and their associated factors among medical students. J. Coll. Physicians Surg. Pak..

[18] Bassols A.M., Okabayashi L.S., Silva A.B., Carneiro B.B., Feijó F., Guimarães G.C., Cortes G.N., Rohde L.A., Eizirik C.L. (2014). First- and last-year medical students: Is there a difference in the prevalence and intensity of anxiety and depressive symptoms?. Rev. Bras. Psiquiatr..

[19] Jadoon N.A., Yaqoob R., Raza A., Shehzad M.A., Zeshan S.C. (2010). Anxiety and depression among medical students: A cross-sectional study. J. Pak. Med. Assoc..

[20] Puthran R., Zhang M.W., Tam W.W., Ho R.C. (2016). Prevalence of depression amongst medical students: A meta-analysis. Med. Educ..

[21] Hope V., Henderson M. (2014). Medical student depression, anxiety and distress outside North America: A systematic review. Med. Educ..

[22] Ahmed I., Banu H., Al-Fageer R., Al-Suwaidi R. (2009). Cognitive emotions: Depression and anxiety in medical students and staff. J. Crit. Care.

[23] Schwenk T.L., Davis L., Wimsatt L.A. (2010). Depression, stigma, and suicidal ideation in medical students. JAMA.

[24] Dahlin M., Joneborg N., Runeson B. (2005). Stress and depression among medical students: A cross-sectional study. Med. Educ..

[25] Matsushita M., Kumano-Go T., Suganuma N., Adachi H., Yamamura S., Morishima H., Shigedo Y., Mikami A., Takeda M., Sugita Y. (2010). Anxiety, neuroticism and oxidative stress. Psychiatry Clin. Neurosci..

[26] Hapuarachchi J.R., Chalmers A.H., Winefield A.H., Blake-Mortimer J.S. (2003). Changes in clinically relevant metabolites with psychological stress parameters. Behav. Med..

[27] Vrublevska J., Trapencieris M., Rancans E. (2018). Adaptation and validation of the Patient Health Questionnaire-9 to evaluate major depression in a primary care sample in Latvia. Nord. J. Psychiatry.

[28] Kroenke K., Spitzer R.L., Williams J.B. (2001). The PHQ-9: Validity of a brief depression severity measure. J. Gen. Intern. Med..

[29] Spitzer R.L., Kroenke K., Williams J.B., Löwe B. (2006). A brief measure for assessing generalized anxiety disorder: The GAD-7. Arch. Intern. Med..

[30] Swinson R.P. (2006). The GAD-7 scale was accurate for diagnosing generalised anxiety disorder. Evid. Based Med..

[31] Vrublevska J., Renemane L., Kivite-Urtane A., Rancans E. (2022). Validation of the generalized anxiety disorder scales (GAD-7 and GAD-2) in primary care settings in Latvia. Front. Psychiatry.

[32] Srivastava R., Batra J. (2014). Oxidative stress and psychological functioning among medical students. Ind. Psychiatry J..

[33] Sivonová M., Zitnanová I., Hlincíková L., Skodácek I., Trebatická J., Duracková Z. (2004). Oxidative stress in university students during examinations. Stress.

[34] Alfthan G. (1984). A micromethod for the determination of selenium in tissues and biological fluids by single-test-tube fluorimetry. Anal. Chim. Acta.

[35] Human Selenoprotein P(SEPP1) ELISA Kit. https://www.cusabio.com/ELISA-Kit/Human-Selenoprotein-PSEPP1-ELISA-kit-102874.html.

[36] Ferreira de Almeida T.L., Petarli G.B., Cattafesta M., Zandonade E., Bezerra O.M.P.A., Tristão K.G., Salaroli L.B. (2021). Association of Selenium Intake and Development of Depression in Brazilian Farmers. Front Nutr..

[37] Portnoy J., Wang J., Wang F., Um P., Irving S.Y., Hackl L., Liu J. (2022). Lower serum selenium concentration associated with anxiety in children. J. Pediatr. Nurs..

[38] Shokati S., Kavian Z., Shahraki M., Afshari M. (2021). The Relationship of Dietary Intake of Zinc, Selenium, and Magnesium and Anthropometric Profiles with Depression in Female Medical Students at Zahedan University of Medical Sciences. J. Nutr. Food Secur..

[39] Conner T.S., Richardson A.C., Miller J.C. (2015). Optimal serum selenium concentrations are associated with lower depressive symptoms and negative mood among young adults. J. Nutr..

[40] Tinggi U. (2008). Selenium: Its role as antioxidant in human health. Environ. Health Prev. Med..

[41] Dogaru C.B., Muscurel C., Duță C., Stoian I. (2023). “Alphabet” Selenoproteins: Their Characteristics and Physiological Roles. Int. J. Mol. Sci..

[42] Moghadaszadeh B., Beggs A.H. (2006). Selenoproteins and their impact on human health through diverse physiological pathways. Physiology.

[43] Saito Y. (2021). Selenium Transport Mechanism via Selenoprotein P-Its Physiological Role and Related Diseases. Front. Nutr..

[44] Amirkhizi F., Khalese-Ranjbar B., Mansouri E., Hamedi-Shahraki S., Asghari S. (2023). Correlations of selenium and selenoprotein P with asymmetric dimethylarginine and lipid profile in patients with polycystic ovary syndrome. J. Trace Elements Med. Biol..

[45] Barchielli G., Capperucci A., Tanini D. (2022). The Role of Selenium in Pathologies: An Updated Review. Antioxidants.

[46] Almadani A.H., Alsubaihi A.A., Alsqabi H.A., Alkathiri M.A., Alassaf M.I., Alagel O.A., Alshowihi S.S., Alolayan M.A. (2024). Comparison of depression and anxiety in first- versus non-first generation Saudi medical students: A cross-sectional study. Medicine.

[47] Nunes J.K.V.R.S., Figueiredo V.M.e.S.d., Santos J.V.S.S., Mendes N.M.L.d.S., Neto J.A.d.F. (2022). Anxiety and depression in medical students: A cross-sectional study. Rev. Med..

[48] Mallaram G.K., Shaik S., Kattula D. (2023). Anxiety, Depression and Stress Among Female Medical Students During the Second Wave of the COVID-19 Pandemic and their Association with Family Functioning, Coping and Personality. Curr. Med Issues.

